# Benefits of Substituting Sitting with Standing and Walking in Free-Living Conditions for Cardiometabolic Risk Markers, Cognition and Mood in Overweight Adults

**DOI:** 10.3389/fphys.2017.00353

**Published:** 2017-06-08

**Authors:** Bernard M. F. M. Duvivier, Nicolaas C. Schaper, Annemarie Koster, Linh van Kan, Harry P. F. Peters, Jos J. Adam, Timo Giesbrecht, Esther Kornips, Martine Hulsbosch, Paul Willems, Matthijs K. C. Hesselink, Patrick Schrauwen, Hans H. C. M. Savelberg

**Affiliations:** ^1^Department Human Biology and Human Movement Sciences, NUTRIM School for Nutrition and Translational Research in Metabolism, Maastricht University Medical Centre+Maastricht, Netherlands; ^2^Division Endocrinology, Department Internal Medicine, CARIM School for Cardiovascular Diseases, Maastricht University Medical Centre+Maastricht, Netherlands; ^3^CAPHRI School for Public Health and Primary Care, Maastricht University Medical Centre+Maastricht, Netherlands; ^4^Department Social Medicine, Maastricht University Medical Centre+Maastricht, Netherlands; ^5^Unilever Research and DevelopmentVlaardingen, Netherlands; ^6^Unilever Research and DevelopmentPort Sunlight, United Kingdom

**Keywords:** exercise, insulin sensitivity, light-intensity physical activity, lipids, sedentary behavior, sitting, standing, walking, http://www.clinicaltrials.gov, NCT02394249.

## Abstract

**Background:** We investigated whether substituting sitting with standing and self-perceived light walking in free-living conditions would improve cardiometabolic risk factors, mood, and cognition in overweight/obese adults.

**Methods:** In a randomized, cross-over study, 24 (m/f: 13/11) sedentary overweight/obese participants (64 ± 7 years, BMI 29 ± 2 kg/m^2^) followed two activity regimens of each 4 days in free-living conditions: “Sit”: sitting 13.5 h/day, standing 1.4 h/day, self-perceived light-intensity walking 0.7 h/day; for “SitLess” these activities lasted 7.6, 4.0, and 4.3 h/day, respectively. Meals were standardized and physical activity was assessed by accelerometry (activPAL). Insulin sensitivity (expressed as Matsuda-index based on an oral glucose tolerance test), circulating lipids, blood pressure, mood (pleasantness and arousal), and cognition were assessed on the morning after the activity regimens. Quality of life and sleep were assessed on the last day of the activity regimens.

**Results:** We observed that AUC (0–190 min) for insulin decreased by 20% after SitLess vs. Sit [10,125 (656) vs. 12,633 (818); *p* = 0.006]. Insulin sensitivity improved by 16% after SitLess vs. Sit [Matsuda-index, mean (SEM): 6.45 (0.25) vs. 5.58 (0.25) respectively; *p* = 0.007]. Fasting triglycerides, non-HDL-cholesterol, and apolipoprotein B decreased by 32, 7, and 4% respectively, whereas HDL-cholesterol increased by 7% after SitLess vs. Sit (all *p* < 0.01). Diastolic blood pressure was lower after SitLess vs. Sit (*p* < 0.05). Pleasantness (as one marker of mood status) after the oral glucose tolerance test was higher after SitLess vs. Sit (*p* < 0.05). There was no significant difference between regimens for cognition, quality of life and sleep.

**Conclusions:** Reducing sitting time in free-living conditions markedly improved insulin sensitivity, circulating lipids, and diastolic blood pressure. Substituting sitting with standing and self-perceived light walking is an effective strategy to improve cardiometabolic risk factors in overweight/obese subjects.

## Introduction

Observational studies suggest that the majority of the Western population spends more than half of the waking day sedentary (Matthews et al., 2008; van der Berg et al., 2016b). Mounting evidence shows an association between a high sitting time and obesity (Levine et al., 2005; Chastin et al., 2015; de Rooij et al., 2016). In addition to the health risks associated with overweight and obesity (Hubert et al., 1983; Mokdad et al., 2003), a sedentary lifestyle has been associated with an increased risk of type 2 diabetes, metabolic syndrome, and premature mortality (Biswas et al., 2015; van der Berg et al., 2016b). This negative consequence of sitting seems to be independent of the time spent in moderate-to-vigorous physical activity (Biswas et al., 2015; van der Berg et al., 2016b). Hence, interventions reducing sitting time may improve cardiometabolic health in these individuals. Indeed, laboratory studies showed beneficial effects on circulating glucose and insulin in overweight and obese adults when sitting was interrupted every 20–30 min with light walking (Dunstan et al., 2012; Bailey and Locke, 2015; Henson et al., 2016). However, as recently pointed out by the American Heart Association, interventions in free-living conditions that reduce sitting time are very scarce (Young et al., 2016).

Apart from its cardiometabolic consequences, obesity has also been associated with an increased risk of mood disorders (McElroy et al., 2004) and reduced cognitive function (Smith et al., 2011). This increased risk may partly originate from obesity related insulin resistance in the brain (Lamport et al., 2009). Vice versa, improvements in insulin sensitivity have been linked to improvements in mood and cognition (Kim and Feldman, 2012; Heni et al., 2015). Several studies have shown that engaging in moderate-to-vigorous physical activity not only improves insulin sensitivity (Wojtaszewski et al., 2000), but also mood (Brown et al., 2009) and cognition (Smith et al., 2010). However, to which extent these beneficial effects also hold true for light-intensity physical activity is unclear.

In the present study, we investigated whether substituting sitting with standing and self-perceived light walking in free-living conditions improved insulin sensitivity and other cardiometabolic risk factors in sedentary overweight/obese individuals. Moreover, we explored whether reducing sitting time also improved mood and cognition.

## Methods

### Participants

Adults aged 40–80 years with a BMI between 25 and 35 kg/m^2^, were recruited through paper advertisements at Maastricht University and through online and newspaper advertisements outside Maastricht University. During screening, every individual performed a 1 day try-out of the SitLess regimen to ensure that the participant was able to carry out the SitLess regimen in free-living conditions. Physical activity was measured during 4 days (including one weekend day) in free-living conditions before the start of the study. Exclusion criteria were more than 2.5 h/week of moderate-to-vigorous physical activity based on self-report, diseases which interfered with physical activities, weight loss (>2 kg) in the last 3 months, alcohol abuse, experimental drug use, use of glucose lowering drugs, corticosteroids, or coumarins or fasting plasma glucose >6.9 mmol/l. Throughout the study, drug administration and usage remained unaltered. All participants provided written informed consent. The study was conducted at Maastricht University between February and September 2015. (www.clinicaltrials.gov, NCT02394249). This study was carried out in accordance with the recommendations of the Local Ethics Committee of the Maastricht University Medical Centre+ with written informed consent from all subjects. All subjects gave written informed consent in accordance with the Declaration of Helsinki. The protocol was approved by the Local Ethics Committee of the Maastricht University Medical Centre+.

### Study design

The primary outcome was Area Under the Curve (AUC) for plasma insulin during an Oral Glucose Tolerance Test (OGTT). Based on an earlier study in healthy subjects with a similar design (Duvivier et al., 2013), the number of subjects required was calculated. Based on mean difference ± SD in AUC for insulin (1257.5 ± 2293.5 mU/l × min) between the two activity regimens and a two-sided alpha of 0.05, we calculated that 21 subjects would be needed to detect a difference of 1,500 mU/l × min between the SitLess and the Sit regimen with a power of 80% using a paired-samples *t*-test. To account for a 15% drop-out after randomization, 25 subjects were included.

### The activity regimens

**Figure 1:**
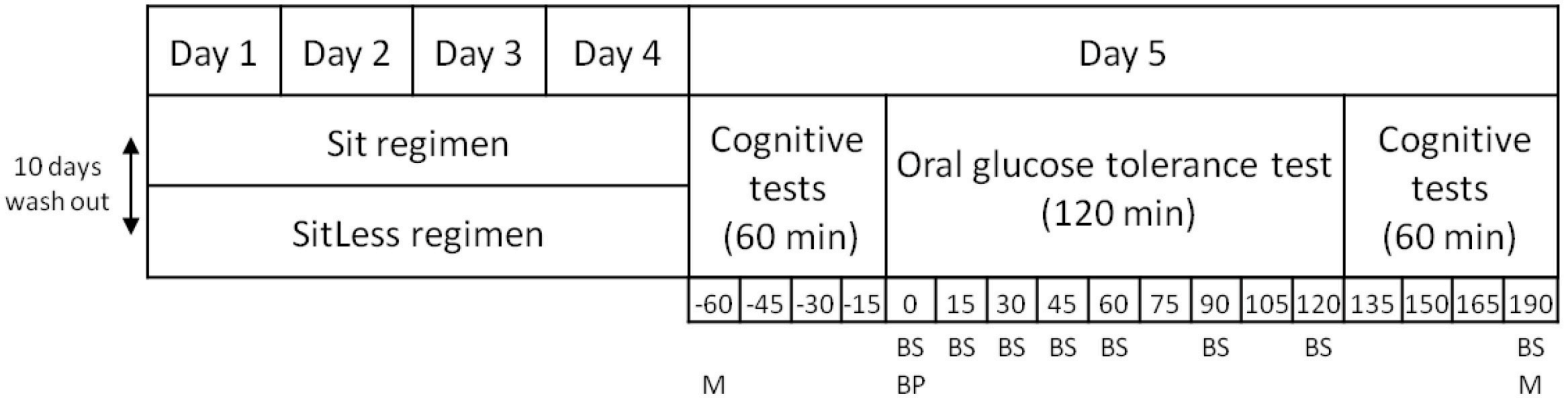
Study design. BP, blood pressure; BS, blood sample; M, mood assessment.

All participants were instructed to follow two activity regimens in free-living conditions, lasting 4 days each (Sit and SitLess). The study had a randomized cross-over design. Randomization was performed by a computer program with a block size of two intervention orders; each pair of included persons received another regimen order. The study design is displayed in Figure 1. During Sit, participants were instructed to restrict walking and standing to ≤ 1 h/day each, spending the remainder of the waking day sitting. During SitLess, participants were instructed to substitute at least 7 h/day of sitting with ≥4 h of self-perceived light walking and ≥3 h of standing; and to interrupt sitting preferably every 30 min with standing/walking bouts. Subjects were instructed to walk at a self-perceived light-intensity. Adherence to these instructions was monitored by accelerometry (see below). There was a wash-out period of at least 10 days between the screening session and the first activity regimen, and between the two activity regimens. During the wash-out, participants were instructed to maintain their habitual pattern of daily life activities, not to perform more than 1 h/week of moderate-to-vigorous physical activity and to consume a maximum of 1 unit/day of alcohol.

### Meal standardization

During the activity regimens, subjects were instructed to adhere to their normal diet. During the first regimen, participants carefully recorded everything they ate, and drank of these consumptions in a diary. These records were returned to the participants who were instructed to consume the same diet during the second activity regimen. Alcohol was not permitted during the activity regimens. In order to achieve identical energy intake and meal composition in the 12 h before the final measurements, participants received identical pre-packaged meals for dinner on the last day of each activity regimen. The pre-packaged meals included a main meal (vegetables, potatoes, and chicken or pork, 409–437 kcal, 11.3–15.8 g fat, 45.0–51.8 g carbohydrates, 20.3–22.5 g protein) and a dessert (yogurt, 150 kcal, 3.8 g fat, 13.1 g carbohydrates, 2.9 g protein). The subjects were instructed to consume this meal at home before 22.00 and to refrain from food or drinks after this meal except for water.

### Assessment of physical activity

Physical activity and posture allocation were measured 24 h/day using an activPAL3 activity monitor (PAL Technologies, Glasgow, Scotland). The monitor was attached waterproof to the skin on the anterior thigh using Tegaderm (3M, St. Paul, Minnesota, USA) at least 1 day before each activity regimen. This accelerometer accurately discriminates between time spent inactive (sitting or lying), standing, walking (Berendsen et al., 2014), and step number (Ryan et al., 2006). Since the activPAL program does not provide sleeping time automatically, sleeping time was determined with a validated algorithm (van der Berg et al., 2016a), which was implemented as a Matlab (Mathworks, Natick, MA) program. Diary data for self-reported physical activity were compared with the activPAL3 data to formulate tailor-made instructions on how to change daily activities after the first and third days of each activity regimen to guarantee optimal compliance to each activity regimen.

### Oral glucose tolerance test

After each activity regimen (day 5), the subjects came to the research center between 8:30 and 9:30 AM after an overnight fast and an OGTT was performed. After an acclimatization period of 10 min, blood pressure was measured three times with an Omron 705IT blood pressure monitor (Omron Healthcare Europe B.V., Hoofddorp, The Netherlands). An i.v. catheter was placed in an antecubital vein for blood sampling. At baseline, blood was sampled for analysis of glucose, insulin, C-peptide, triglycerides, free fatty acid (FFA) levels, total cholesterol, high-density-lipoprotein (HDL) and low-density-lipoprotein (LDL) cholesterol, non-HDL-cholesterol, apo A-I, and B100. After ingestion of 75 g of glucose in water (200 ml in total), blood samples were drawn for glucose, insulin and C-peptide levels at 15, 30, 45, 60, 90, 120, and 190 min. Blood samples were stored at –80°C until analysis after the end of the study. Insulin and C-peptide were measured using a Human Insulin Specific RIA kit (HI-14K, Millipore) and a Human C-peptide RIA kit (HCP-20K, Millipore) respectively. Radioactivity was count on a 2,470 Automatic Gamma Counter (Perkin Elmer). Plasma glucose, total cholesterol, HDL-cholesterol, triglycerides, free fatty acids, apo A-I, and apo B100 were spectrophotometrically analyzed on the ABX Pentra 400 (Horiba) and free glycerol on a Cobas Fara (Roche). Plasma samples were precipitated with 1/10 volume of sulfosalicylic acid, placed on ice for 25 min, and then centrifuged at maximal speed. Free glycerol was measured in the supernatant. LDL-cholesterol was calculated using the Friedewald formula (Friedewald et al., 1972). Non-HDL-cholesterol was calculated as total cholesterol minus HDL-cholesterol.

### Mood and cognition

Cognitive performance and mood were measured before and after the OGTT, based on the principle that by applying a challenge (in this case the glucose load), one might be better able to measure the impact of interventions, such as physical activity (van Ommen et al., 2014). Mood was assessed with the Affect Grid test; which is a 19 × 19 single-item measure, assessing the self-reported degree of pleasantness and arousal of the participants (Russell et al., 1989). Verbal memory (immediate and delayed) was assessed with Rey's Verbal Learning Test (Van der Elst et al., 2005), executive function was assessed with the Trail Making Test (Bowie and Harvey, 2006; Oosterman et al., 2010), and attention with the Attention Network Test covering the dimensions alerting, orienting, and executive function (Fan et al., 2005). On day 4 of each activity regimen, quality of life was assessed with a 32-item questionnaire of Gill et al. (2013) and sleep quality was assessed with the 10-item Pittsburgh Sleep Quality Index (Buysse et al., 1989).

### Data processing and statistical analysis

The AUC over a period of 190 min after glucose ingestion was calculated for insulin and C-peptide using the trapezoidal rule approach (Brouns et al., 2005). For glucose, the positive incremental area under the curve (iAUC) was calculated as the AUC above the baseline level. Insulin sensitivity, expressed as the Matsuda index, was calculated based on glucose and insulin values during the first 120 min of the OGTT (Matsuda and DeFronzo, 1999).

All statistical calculations were performed using SAS (version 9.4, Cary, NC, USA) or IBM SPSS (version 21, Armonk, NY, USA). The differences in blood related outcome parameters and blood pressure between the activity regimens were analyzed using linear mixed model analyses including the activity regimen, order of the activity regimens and baseline characteristics as fixed factors. Since associations between sedentary behavior and cardiometabolic risk factors have previously been reported to be stronger in women (Owen et al., 2010), sex was added to the model as a co-variate. For the AUC and iAUC calculations, values at *t* = 0 were added as fixed factor to the model. For the mood scores (arousal and pleasantness), the linear mixed model included time as a categorical variable including its interaction with activity regimen, values at *t* = 0 and order of testing. The residual error structure was described with an ARH(1)-covariance matrix to handle variance heterogeneity at the time points. Similar analyses were performed for the cognitive parameters. For some subjects, part of the mood and cognition data was excluded from the statistical analysis due to technical errors during the mood and cognition tests. A log transformation was performed for glucose, insulin, C-peptide, and diastolic blood pressure. Numerical variables are presented as mean ± SD for baseline characteristics, mean ± standard error (SEM) for cardiometabolic risk factors and LSmeans (95% CI) for mood and cognition. *P*-values ≤ 0.05 were considered statistically significant.

## Results

### Subjects

After screening 25 subjects (13 men, 12 women) were included. Before completing the protocol, one female participant withdrew because of cholangitis. The remaining 24 participants had a mean age of 64 ± 7 years and BMI of 29.4 ± 2.3 kg/m^2^ (Table 1). Female participants had a significantly higher BMI and lower age and height than male participants. Five participants were using cholesterol lowering drugs (statins) and six participants were using blood pressure lowering drugs (3 angiotensin receptor blockers, 2 calcium channel blockers, 1 ACE-inhibitor, 1 beta blocker).

**Table 1:** Subject characteristics.

**Variables**	**Total**	**Men**	**Women**
N	24	13	11
Age (years)^*^	64 ± 7	67 ± 2	59 ± 9
Height (m)^*^	1.72 ± 0.08	1.76 ± 0.07	1.68 ± 0.07
Weight (kg)	87.1 ± 9.7	88.3 ± 9.6	85.7 ± 10.1
BMI (kg/m^2^)^*^	29.4 ± 2.3	28.5 ± 1.7	30.5 ± 2.5
Waist circumference (cm)^†^	104 ± 10	104 ± 8	103 ± 11
Systolic blood pressure (mmHg)	143 ± 17	148 ± 15	136 ± 18
Diastolic blood pressure (mmHg)	83 ± 9	83 ± 9	82 ± 8
Fasting glucose (mmol/l)	5.5 ± 0.6	5.5 ± 0.5	5.4 ± 0.7

*Data are presented as mean ± SD*.

* p < 0.05 for sex;

† *n = 12 for men; n = 10 for women*.

### Insulin sensitivity

**Figure 2:**
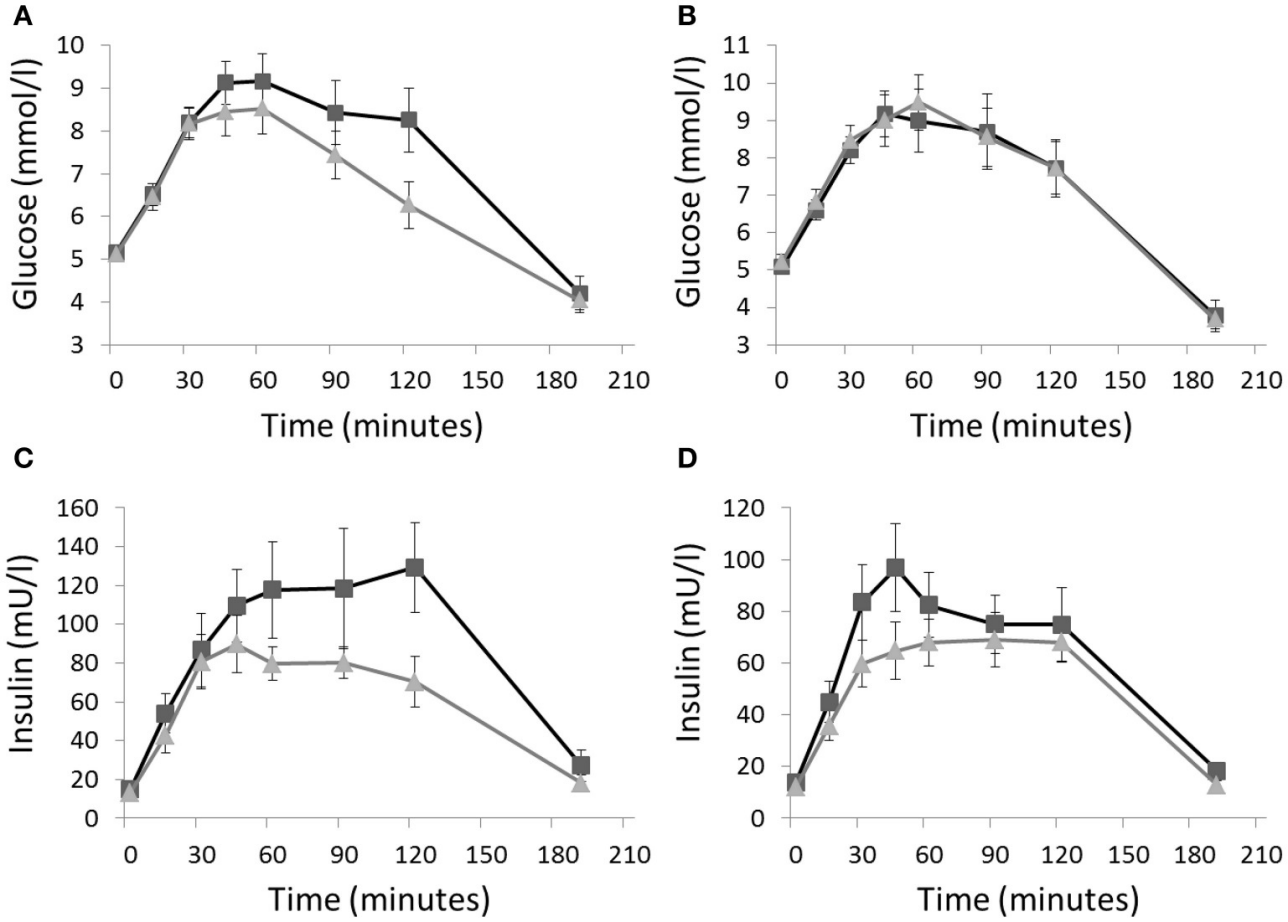
Glucose and insulin responses to an oral glucose tolerance test on the morning after the Sit (■) and SitLess (▴) regimens for respectively women **(A,C)** and men **(B,D)**. iAUC for glucose in women was lower after SitLess vs. Sit (*p* = 0.006), but not in men (*p* = 0.266). AUC for insulin was significantly lower after SitLess vs. Sit in men and women (*p* = 0.006). Means and standard error bars are presented.

**Figure 3:**
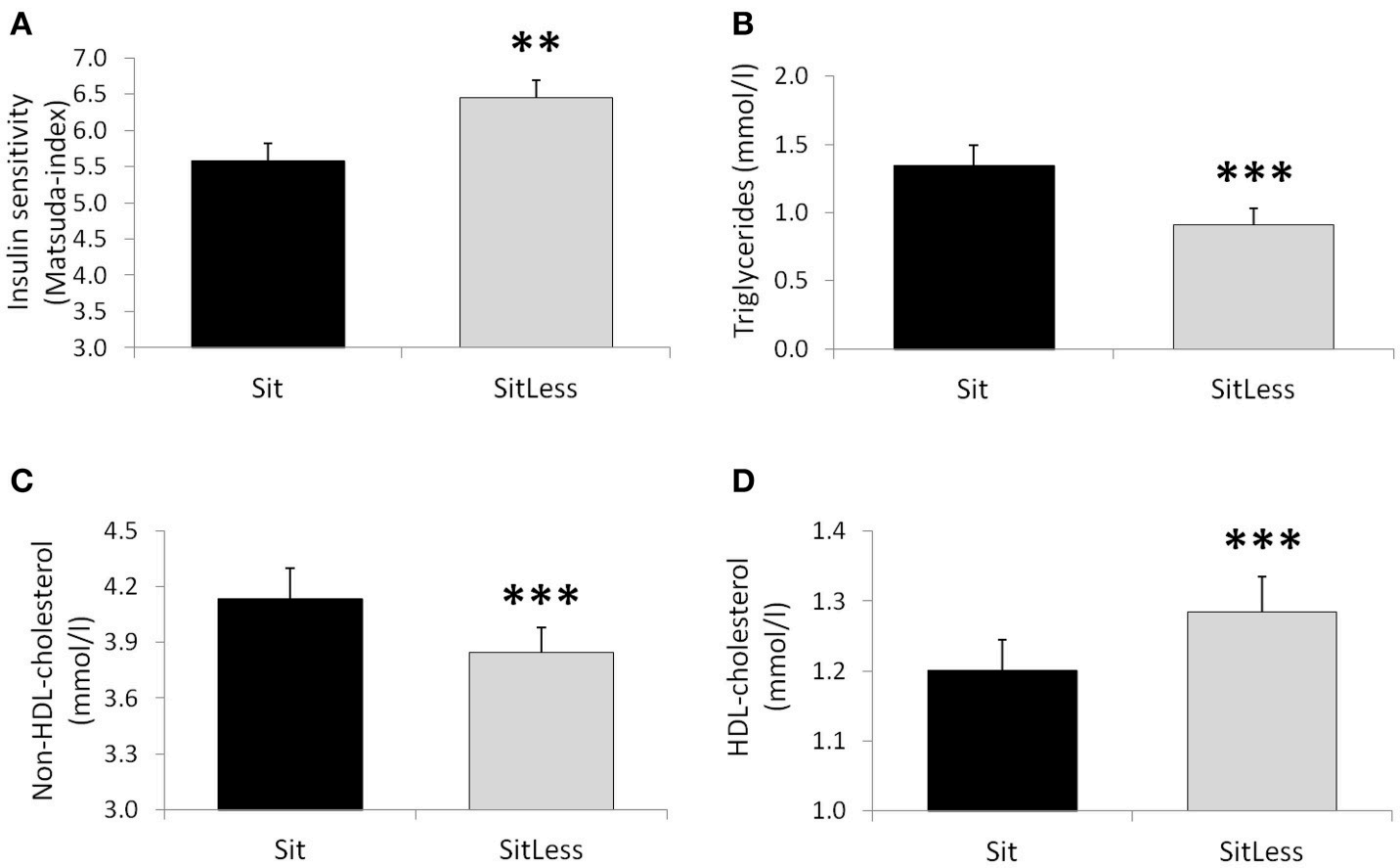
Insulin sensitivity (Matsuda-index; **A**), triglycerides **(B)**, non-HDL-cholesterol **(C)** and HDL-cholesterol **(D)** on the morning after the activity regimens. Means and standard error bars are presented. ^**^p < 0.01; ^***^p < 0.001.

After the activity regimens, there was no significant difference in the iAUC for glucose between Sit and SitLess (Table 2). AUC for insulin (Table 2; Figure 2) decreased by 20% after SitLess vs. Sit [mean (SEM): 10,125 (656) vs. 12,633 (818); *p* = 0.006]. As a result, insulin sensitivity (Figure 3) was 16% higher after SitLess vs. Sit [Matsuda-index: 6.45 (0.25) vs. 5.58 (0.25) respectively; *p* < 0.001]. The AUC for C-peptide was 7% lower (*p* = 0.032) after SitLess vs. Sit. In subgroup analyses the iAUC for glucose in women was lower after SitLess vs. Sit (–32%; *p* = 0.006), while no significant difference was observed in men (+14%; *p* = 0.266). No sex-differences were observed in Matsuda-index and AUC for insulin and C-peptide.

**Table 2:** Cardiometabolic risk factors.

**Variables**	**Sit**	**SitLess**	***P*-value**
Fasting glucose (mmol/l)	5.1 (0.1)	5.2 (0.1)	0.153
Glucose iAUC (mmol/l × min)	367 (40)	325 (36)	0.159
Fasting insulin (mU/l)	13.2 (1.0)	11.4 (0.9)	**0.003**
Insulin AUC (mU/l × min)	12,633 (818)	10,125 (656)	**0.006**
Fasting C-peptide (ng/ml)	1.75 (0.12)	1.53 (0.10)	< **0.001**
C-peptide AUC (ng/ml × min)	1,187 (42)	1,104 (39)	**0.032**
Apolipoprotein A-I (g/l)	1.45 (0.03)	1.46 (0.03)	0.366
Apolipoprotein B100 (g/l)	1.07 (0.04)	1.03 (0.03)	**0.007**
Free fatty acids (mmol/l)	0.59 (0.03)	0.69 (0.04)	**0.014**
Free glycerol (mmol/l)	0.14 (0.01)	0.16 (0.01)	0.062
Systolic BP (mmHg)	138 (4)	137 (3)	0.729
Diastolic BP (mmHg)	81 (1)	79 (1)	**0.043**
HR (beats/min)	64 (2)	62 (2)	0.170

*Data are presented as mean (SEM). BP, blood pressure; HR, heart rate; iAUC, incremental AUC. Bold values indicate p < 0.05*.

### Circulating lipids and blood pressure

After the activity regimens, triglycerides, total cholesterol, non-HDL-cholesterol, and apolipoprotein B were lower following SitLess vs. Sit by 32, 4, 7, and 4% respectively (all *p* < 0.01; Table 2; Figure 3). HDL-cholesterol was 7% higher (*p* < 0.001) and FFA levels were 17% higher (*p* = 0.014) after SitLess vs. Sit. Diastolic blood pressure was lower after SitLess vs. Sit (*p* = 0.043). Systolic blood pressure, heart rate, apolipoprotein A, and free glycerol did not differ significantly between Sit and SitLess. In subgroup analyses, the magnitude of triglyceride attenuation was significantly greater in men (−38%; *p* < 0.001) than in women (−27%; *p* < 0.001) after SitLess vs. Sit. No sex-differences were observed in the other lipid variables, blood pressure, and heart rate.

### Mood and cognition

**Figure 4:**
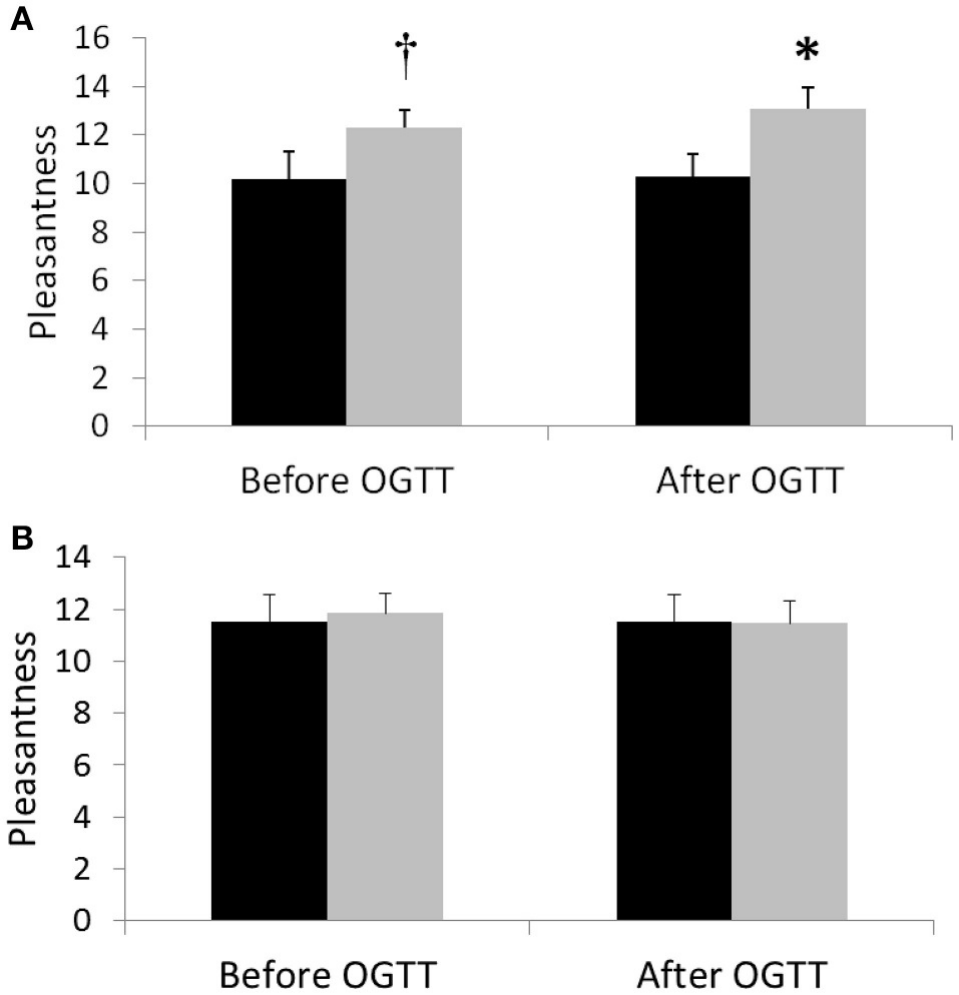
Pleasantness 1 day after the SitLess (gray) and Sit (black) regimens in women **(A)** and men **(B)**. Pleasantness was measured before (−60 min) and 190 min after administering an oral glucose drink (OGTT). ^*^p < 0.05; †*p* = 0.059.

After the activity regimens, we performed measurements of mood and cognition both before the OGTT in the fasted state, as well as after an OGTT. Before the OGTT, pleasantness was not different between the activity regimens for the total group, although a non-significant improvement (*p* = 0.059) was observed in women after SitLess vs. Sit (estimated change 2.20, 95% CI: –0.08–4.48; *n* = 10; Figure 4). After the OGTT, pleasantness was significantly higher after SitLess vs. Sit (1.67; CI: 0.09–3.25; *n* = 21) in the total group; this could mainly be explained by a significant difference in pleasantness in the female subjects after SitLess vs. Sit (2.80; CI: 0.52–5.08; *n* = 10). There was no significant difference in the alerting, orienting and executive dimensions of attention between the activity regimens, neither before nor after the OGTT. Only in female subjects after the OGTT, alertness was significantly higher (–14.8 ms; CI: –29.1 to –0.5; *n* = 11) after SitLess vs. Sit. There were no significant differences in memory, executive function, quality of life, and sleep between the activity regimens.

### Physical activity and diet

At baseline (before the start of the study), time spent sitting/lying was 18.4 ± 1.6 h/day, walking 1.8 ± 0.6 h/day and standing 3.8 ± 1.2 h/day. During the activity regimens, time spent sitting, walking, and standing in free-living conditions were successfully altered in accordance with the protocol (Table 3). During SitLess, time spent sitting (7.6 h/day), walking (4.0 h/day) and standing (4.3 h/day) were significantly different than during Sit (13.5 h/day sitting, 0.7 h/day walking, and 1.4 h/day standing). Sedentary bouts >30 min were significantly lower during SitLess (3.9 bouts) compared to Sit (8.5 bouts). Sleeping time was comparable between SitLess (8.0 h/day) and Sit (8.2 h/day). Energy intake did not differ significantly between the activity regimens, neither did the percentage macronutrients consumed (Table 3).

**Table 3:** Physical activity and diet.

**Variables**	**Sit**	**SitLess**	***P*-value**
**PHYSICAL ACTIVITY**			
Sitting (h/day)	13.5 (0.2)	7.6 (0.3)	< **0.001**
Standing (h/day)	1.4 (0.1)	4.0 (0.2)	< **0.001**
Walking (h/day)	0.7 (0.1)	4.3 (0.1)	< **0.001**
Sleeping (h/day)	8.2 (0.2)	8.0 (0.2)	**0.027**
Steps/day (n)	3,228 (187)	24,626 (509)	< **0.001**
Sedentary bouts >30min (n/day)	8.5 (0.3)	3.9 (0.2)	< **0.001**
**DIET**			
Energy intake (kcal/day)	1,930 (77)	1,943 (94)	0.669
Carbohydrates (%)	47.3 (1.4)	47.9 (1.3)	0.422
Protein (%)	17.8 (0.7)	18.0 (0.8)	0.491
Fat (%)	34.8 (1.3)	34.1 (1.2)	0.205
Saturated fat (%)	13.3 (0.5)	13.3 (0.5)	0.723

*Daily activities (activPAL data) and diet (diary data) during each activity regimen. Data are presented as mean (SEM). Bold values indicate p < 0.05*.

## Discussion

In the current study, we observed that substituting sitting with standing and self-perceived light walking improved insulin sensitivity, circulating lipids and diastolic blood pressure in overweight/obese subjects. Interestingly, while other studies reported positive effects on plasma glucose and insulin during interruptions in sitting time (Dunstan et al., 2012; Peddie et al., 2013; Blankenship et al., 2014), we observed improvements in insulin sensitivity 1 day after the SitLess intervention, suggesting that this beneficial effect persists into the next day. These results build on our previous findings in young healthy (Duvivier et al., 2013) and diabetic adults (Duvivier et al., 2017), strongly suggesting that light activities are a very effective measure to improve insulin sensitivity.

In addition to the effects on insulin sensitivity, we observed major improvements in circulating lipids after the SitLess regimen. Interestingly, the magnitude of the changes was comparable or larger than observed with exercise. Thus, exercise training has consistently been shown to increase HDL-cholesterol levels; a meta-analysis of RCT's reported an average 0.06 mmol/l increase when adhering to the exercise (~150 min/week) guidelines (Kodama et al., 2007). In comparison, the SitLess regimen in our study resulted in an HDL-cholesterol increase of 0.08 mmol/l. To our knowledge, we are the first to show an increase in HDL-cholesterol after an acute sit less intervention. Hence, light activities such as standing and light walking seem to be effective in increasing HDL-cholesterol levels to a similar degree as exercise. In line with this result, we also observed a profound reduction in triglycerides (−32%) as well as a reduction in non-HDL-cholesterol, apolipoprotein B and diastolic blood pressure after the SitLess regimen, suggesting that reducing sitting time improves the cardiometabolic profile even further.

Our results may be especially important for sedentary overweight/obese subjects as these individuals are at high risk of developing cardiometabolic disease (Hubert et al., 1983; Mokdad et al., 2003). It was recently observed that each additional hour of sitting increased the odds for type 2 diabetes and metabolic syndrome by 22 respectively 39% (van der Berg et al., 2016b). Engaging in structured exercise as a countermeasure is a challenge for many individuals. Less than 5% of the population adheres to the exercise guidelines (Troiano et al., 2008) and physical activity has been reported to be even lower in people who are obese (Levine et al., 2005; de Rooij et al., 2016). Hence, reducing sedentary behavior might be a more feasible alternative. Strategies to reduce sitting time are generally considered less demanding than structured exercise programs and hence are more likely to have long term compliance (Martin et al., 2015). Our observations suggest that substituting sitting with light activities may have major cardiometabolic benefits and could potentially reverse the adverse cardiometabolic risk that is associated with sedentary behavior.

We observed sex-differences in glucose tolerance between the activity regimens. In comparison to the Sit regimen, SitLess lowered glucose iAUC levels significantly in female participants (−32%), but did not differ significantly in male participants (+14%). In contrary, the magnitude of triglyceride attenuation was significantly greater in men than in women after the SitLess regimen. These differences could not be explained by sex-differences in physical activity or diet during the activity regimens. The sex-differences for glucose are in line with a recent intervention study in obese adults with type 2 diabetes (Dempsey et al., 2016), in which postprandial glucose levels were also significantly lower in women (–58%) than in men (–26%) when sitting was interrupted with self-perceived light-intensity walking. It is possible that sex-differences in adipose and lean muscle mass can explain our observations; however, these variables were not measured in our study. Further studies should shed light on the underlying mechanisms explaining these possible sex-differences.

We observed that insulin sensitivity improved after the SitLess intervention, which is consistent with previous findings reporting an upregulation of the insulin signaling pathway after 3 days of interrupting sitting with light-intensity walking (Bergouignan et al., 2016). The decrease in triglyceride levels after the SitLess regimen could possibly be explained by enhanced lipoprotein lipase activity; thus, physical activity increases lipoprotein lipase mRNA and typically peaks ≥4 h after physical activity (Seip et al., 1997) and our results suggest that light-intensity activity may already be sufficient to elicit such effect. An inverse relationship is known to exist between the triglycerides and HDL-cholesterol levels. During exercise, the action of cholesterol esther transfer protein (CETP) produces triglyceride-rich HDL2 particles, resulting in an HDL-cholesterol increase (Zhang et al., 2013). Therefore, the reduction in triglycerides could have contributed to the increase in HDL-cholesterol following the SitLess regimen. We also observed, in line with previous exercise (Bilet et al., 2011) and light-intensity activity studies (Henson et al., 2016; Duvivier et al., 2017), that FFA levels were higher following the SitLess regimen. This increase in FFA levels was accompanied by a non-significant (*p* = 0.06) increase in free glycerol and may therefore result from elevation of adipose tissue lipolysis to fuel muscle for contractile activity (Jocken and Blaak, 2008).

In addition to cardiometabolic risk factors, we also explored the effects of reducing sitting time on mood and cognition. We observed significant improvements following the SitLess regimen in pleasantness after the OGTT in women. This result is in line with a recent study that observed sex-differences in mood response to exercise (McDowell et al., 2016). Also, alertness was somewhat higher after the OGTT in women following the SitLess regimen. Further research is necessary to assess the robustness of these sex-differences observed.

Strengths of our study include the cross-over randomized design in free-living conditions. Also, adherence to the activity regimens was according to the protocol which was measured 24 h/day by a validated activity monitor. Diet was standardized and energy intake and macronutrient percentage did not differ between the activity regimens. However, the study was not powered to detect differences in mood and cognition or to detect sex-differences. Hence, these findings should be considered exploratory and need replication. This study was a proof-of-concept study of short duration, and as a result the number of steps during the SitLess regimen (about 25,000 steps/day) was well above what is on average observed in a healthy population (about 6,000–13,000 steps/day; Tudor-Locke and Myers, 2001). Thus, the next logical step is to perform dose-response studies to inform about the optimal duration and pattern of time spent standing and light walking and its feasibility in real life circumstances. It also needs to be established whether the acute changes observed in this study persist on the longer-term.

## Conclusion

In conclusion, our study suggests that substituting sitting with standing and self-perceived light walking is a very effective strategy to improve insulin sensitivity, circulating lipids, and diastolic blood pressure in sedentary overweight/obese subjects. Particularly for overweight/obese individuals, these results may be important as strategies to reduce sitting time are generally considered less demanding than structured exercise programs.

## Author contributions

BD, HS, PS, HP, JA, Lv, and TG conceived and designed the experiments. BD and LV performed the experiments, enrolled patients, and performed the data collection. BD, EK, MH and PW performed the data analysis. BD, HS, PS, HP, JA, TG, NS, AK, and MH were involved data interpretation. BD wrote the first draft of the manuscript. All authors contributed to the writing of the manuscript and approved the final version of the manuscript.

### Conflict of interest statement

The authors declare that this study received funding from Unilever Research & Development. This funder was involved in the study design, data analysis and interpretation of the data. Authors HPFP and TG were employed by Unilever Research & Development. BMFMD was funded by a Kootstra Talent Fellowship from the Centre for Research Innovation, Support and Policy of Maastricht University Medical Centre+ to BMFMD. We acknowledge additional support from the Netherlands Cardiovascular Research Initiative: an initiative with support of the Dutch Heart Foundation (CVON2014-02 ENERGISE). There are no patents, products in development or marketed products to declare. The other authors declare that the research was conducted in the absence of any commercial or financial relationships that could be construed as a potential conflict of interest.

## Abbreviations

OGTT: oral glucose tolerance test.
